# Machine learning based ultrasomics noninvasive predicting EGFR expression status in hepatocellular carcinoma patients

**DOI:** 10.3389/fmed.2024.1483291

**Published:** 2024-11-19

**Authors:** Yujing Ma, Shaobo Duan, Shanshan Ren, Didi Bu, Yahong Li, Xiguo Cai, Lianzhong Zhang

**Affiliations:** ^1^Henan University People’s Hospital, Henan Provincial People’s Hospital, Zhengzhou, China; ^2^Department of Health Management, Henan Provincial People’s Hospital, Zhengzhou, China; ^3^Department of Ultrasound, Henan Provincial People’s Hospital, Zhengzhou, China; ^4^Zhengzhou University People’s Hospital, Henan Provincial People’s Hospital, Zhengzhou, China; ^5^Henan Rehabilitation Clinical Medical Research Center, Henan Provincial People’s Hospital, Zhengzhou, China; ^6^Henan International Joint Laboratory of Ultrasonic Nanotechnology and Artificial Intelligence in Precision Theragnostic Systems, Henan Provincial People’s Hospital, Zhengzhou, China

**Keywords:** hepatocellular carcinoma (HCC), machine learning, ultrasomics, epidermal growth factor receptor (EGFR), lenvatinib

## Abstract

**Objective:**

To investigate the ability of ultrasomics to noninvasively predict epidermal growth factor receptor (EGFR) expression status in patients with hepatocellular carcinoma (HCC).

**Methods:**

198 HCC patients were comprised in the study (*n* = 138 in the training dataset and *n* = 60 in the test dataset). EGFR expression was detected by immunohistochemistry. Ultrasomics features from gray-scale ultrasound images were extracted. Intra-class correlation coefficient (ICC) screening, variance filtering, mutual information method, and extreme gradient boosting (XGboost) embedding method were applied for selecting the best features. Random forest (RF), XGBoost, support vector machine (SVM), decision tree (DT), and logistic regression (LR) 5 machine learning algorithms were used to construct clinical models, ultrasomics models, and clinical-ultrasomics combined models, respectively. Area under the receiver operating characteristic curve (AUC), sensitivity, specificity, accuracy, decision curve analysis (DCA), and calibration curve were used to assess the predictive performance of the model.

**Results:**

In 198 patients, high EGFR expression was observed in 100 patients and low EGFR expression was observed in 98 patients. The RF machine learning ultrasomics model was found to perform well, with the AUC of the training and test dataset being 0.929 (95%CI, 0.874–0.966) and 0.807 (95%CI, 0.684–0.897) respectively, the sensitivity being 0.843 and 0.767 respectively, the specificity being 0.857 and 0.800 respectively, and the accuracy being 0.850 and 0.783, respectively. The predictive performance of the combined model established by integrating ultrasomics features and clinical baseline characteristics was improved, with the AUC, sensitivity, specificity, and accuracy of the RF machine learning combined model for the training and test dataset reaching 0.937 (95%CI, 0.884–0.971), 0.822 (95%CI, 0.702–0.909); 0.857, 0.833; 0.857, 0.800; 0.857, 0.817, respectively.

**Conclusion:**

To predict the status of EGFR expression in HCC patients, the ultrasomics model and combined model created by five machine learning algorithms can be utilized as efficient and noninvasive techniques, and the ultrasomics model and combined model established by RF classifier have the best predictive performance.

## 1 Introduction

Hepatocellular carcinoma (HCC) is one of the three leading cancers with the lowest survival rates worldwide (1). In China, liver cancer is burdened and has an insidious onset, and most HCC patients are in advanced stages at presentation, particularly those with cirrhosis or severe liver fibrosis, often losing the opportunity for surgical resection (2). Tyrosine kinase inhibitors (TKIs) and other systemic treatments were made the preferred choice for patients with advanced hepatocellular carcinoma (aHCC) (3, 4). However, the molecular biological and genetic changes during the division of cancer cells endowed HCC with heterogeneous characteristics (5, 6), which affected the therapeutic effects and prognosis of the patients (7).

Epidermal growth factor receptor (EGFR) is located on the cell membrane surface and is a receptor for cell proliferation and signal transduction, and its expression status is related to tumor progression and prognosis (8, 9). EGFR high expression (EGFR^high^) status activates more downstream signaling pathways and promotes proliferation, metastasis and invasiveness of tumor cells, resulting in poor tumor prognosis (10, 11). EGFR is highly expressed in 40–70% of HCC patients, and it has been shown that HCC patients with EGFR^high^ have a poor prognosis and have a shorter survival time than those with low EGFR expression (EGFR^low^) (12, 13). A recent study published in *Nature* found that HCC patients with EGFR^high^ were more likely to develop resistance to TKIs, particularly lenvatinib (12). Only in HCC patients with EGFR^high^, lenvatinib induces the feedback activation of EGFR and its downstream PAK2-ERK5 signaling pathway by inhibiting FGFR and downstream ERK1/2 (14), and simultaneously activates the downstream signaling pathway MEK1/2-ERK1/2, which is common with FGFR, resulting in strong proliferation ability of HCC cells while lenvatinib was administered. EGFR inhibitors effectively blocked feedback activation, and combined with Lenvatinib, produced synergistic antitumor effects, indicating that HCC patients with EGFR^high^ could benefit from this combination (12, 15). Thus, prediction of EGFR expression status not only allows assessment of HCC prognosis, but also enables precise treatment strategies for risk stratification of patients.

EGFR expression requires immunohistochemical detection by surgical resection specimens or biopsies. However, such invasive and less reproducible modality is not suitable for patients with aHCC. Radiomics is a specific algorithm that performs feature extraction and deep mining of standard medical images not only quantifies image features, but also analyzes the molecular phenotype of tumor cells to explore tumor heterogeneity in a non-invasive and reproducible manner (16, 17). Previous researches have reported that computed tomography (CT), magnetic resonance imaging (MRI) and Ultrasound (US) based on radiomics features have the ability to noninvasively characterize biomarkers such as cytokeratin 19 (CK19), vascular endothelial growth factor receptor (VEGFR), and P53 and have achieved promising predictive results (18–20). Up to now, there are few reports on the use of radiomics features to predict EGFR expression in patients with HCC. Since ultrasound is non-invasive, non-radiative, highly repeatable, and reasonably priced, it is one of the most often used techniques for liver testing (20). Thus, the current study is intended to investigate the value of ultrasomics features based on gray-scale ultrasound images for noninvasive prediction of EGFR expression status in patients with HCC, thus providing more objective evidence for precise treatment of aHCC.

## 2 Materials and methods

### 2.1 Study population

735 HCC patients who underwent surgical resection in Henan Provincial People ‘s Hospital from January 2021 to December 2023 were retrospectively analyzed. Inclusion criteria (1) pathological diagnosis of HCC; (2) liver ultrasound examination within 4 weeks before surgery; (3) complete ultrasound and clinical image data. Exclusion Criteria (1) previous treatment with local, systemic or liver transplantation; (2) having tumors in other organs; (3) poor image quality, incomplete lesion display. Finally, 198 HCC patients were included in the study. These 198 patients were randomly stratified (7:3) into a training dataset (*n* = 138) and a test dataset (*n* = 60). The training dataset was processed for imbalanced dataset using the Synthetic Minority Over-sampling Technique (SMOTE) (21).

**Figure 1:**
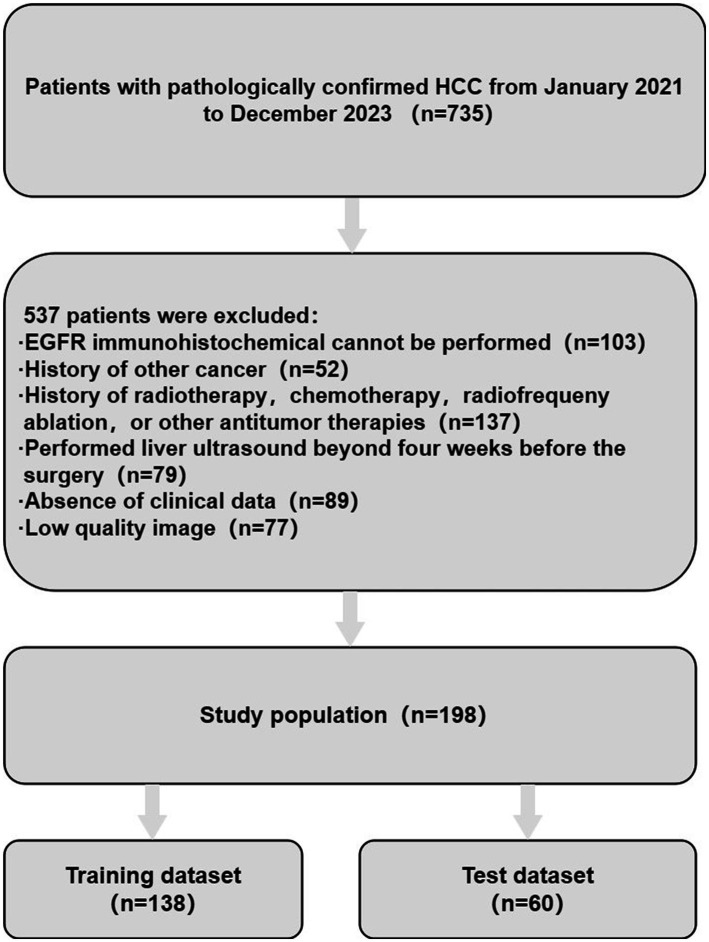
The patients were screened and enrolled according to the established exclusion criteria.

Age, gender, maximum tumor diameter, tumor number, Child-Pugh(A/B/C), cirrhosis (yes/no), HbsAg/HbcAb (positive/negative), portal hypertension (yes/no), Edmondson-steiner grade, alanine aminotransferase (ALT), aspartate aminotransferase (AST), total bilirubin (TBIL), glutamyl transpeptidase (GGT), serum alpha-fetoprotein (AFP), neutrophil-to-lymphocyte ratio (NLR) and other clinical data were derived from medical records. A flow chart of patient selection was shown in Figure 1.

### 2.2 EGFR immunohistochemical analysis

Liver cancer was surgically excised from all patient, regarding the preparation of the immunohistochemical sections provided in Supplementary material 1.

**Figure 2:**
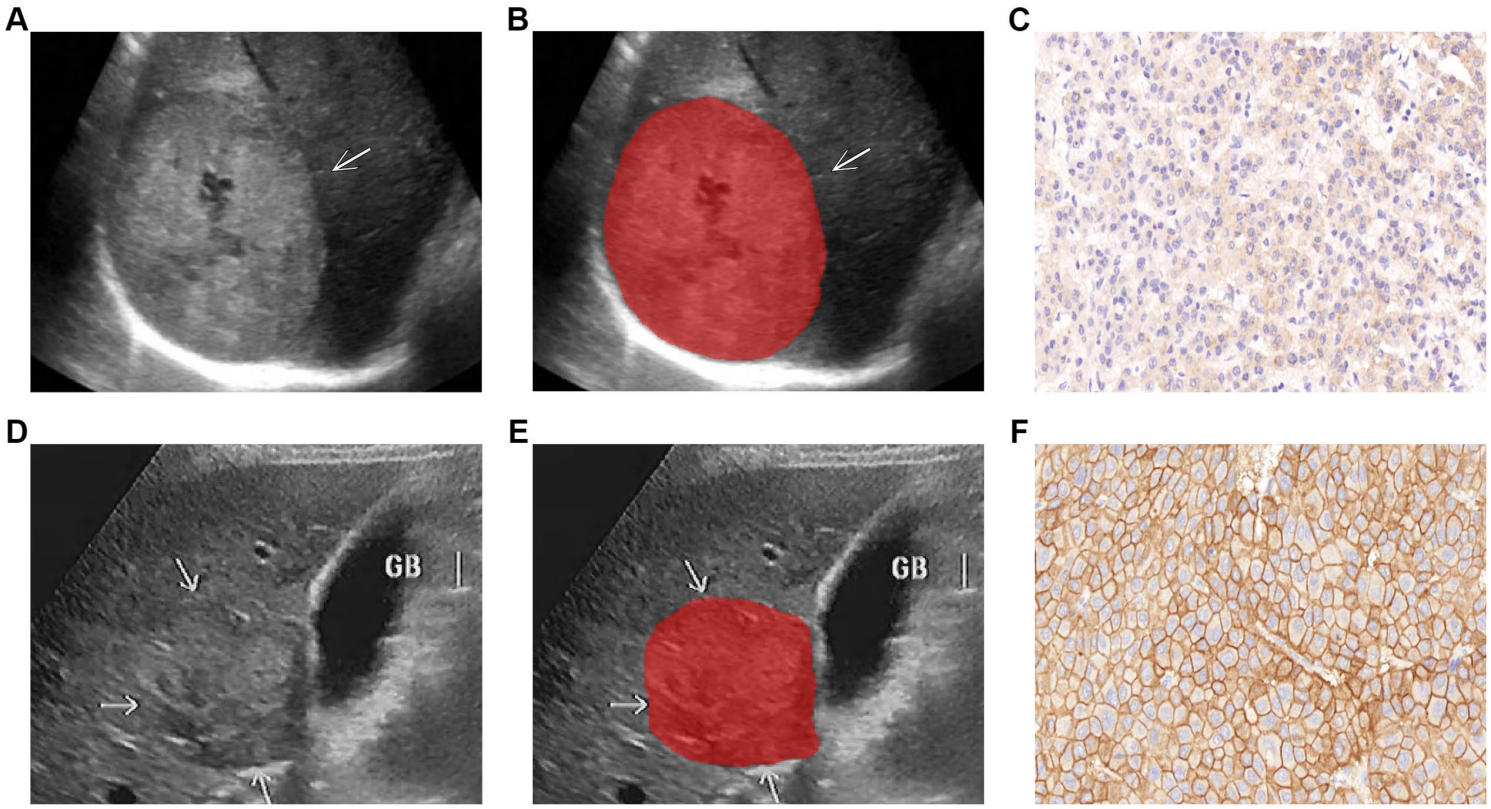
Representative images of lesion segmentation (arrow pointing) and corresponding pathological images of two HCC patients. **(A–C)** Show the gray-scale ultrasound image, lesion segmentation image, and EGFR^low^ pathological image of a 63-year-old male patient (H<200); **(D–F)** Show the gray-scale ultrasound image, lesion segmentation image, and EGFR^high^ pathological image of a 55-year-old male patient (H ≥ 200).

Without knowledge of the patient ‘s information, two observers analyzed the membrane staining intensity of each section and the percentage of number cells at various staining intensities under a light microscope with scores calculated by the H- score formula, and any disagreement assessed by a third observer. Staining intensity was graded into four grades: 0 as no staining; 1+ as weak staining (light brown membrane staining); 2+ as moderate staining (between 1+ and 3+); and 3+ as strong staining (dark brown linear membrane staining) (22). H-score formula: 1× (% of 1+ cells) + 2 × (% of 2+ cells) + 3 × (% of 3 + cells) (23). The score was 0–300 points and the threshold was set at 200 points, and HCC patients were divided into low-expression (H < 200 points) and high-expression (H ≥ 200 points) groups according to score (12, 22) (Figures 2C,F).

### 2.3 Image acquisition

Image scans were performed by physicians with over 8 years of abdominal ultrasound experience, and ultrasound image imaging features were qualitatively assessed: (1) Lesion margin (clear/unclear); (2) Lesion echo (Hypo/Iso/hyper-echoic). Tumor images of the largest diameter were stored in Digital Imaging and Communications in Medicine (DICOM) format for further study (as shown in Figures 2A,D). Ultrasonographic parameters were presented in Supplementary material 2.

### 2.4 Image segmentation

HCC lesions were defined as regions of interest (ROIs). The ITK-SNAP program (version 3.8.0, Figure, www.itksnap.org) was used to import all ultrasound pictures, the delineation process was performed independently by two sonographers with 10 and 15 years of experience in the field, confirmed by a senior sonographer (with 25 years of expertise), and the clinical data about the patient was blinded by the three physicians to avoid differences between and within observers affecting the results. Thirty ultrasound images were randomly selected to assess interobserver reproducibility. The intra-class correlation coefficient (ICC) was used to evaluate the characteristic, and features with ICC ≥ 0.80 were defined as having good agreement (20, 24) to improve the repeatability of features. Segmented images of the lesions were shown in Figures 2B,E.

### 2.5 Feature extraction

Before feature extraction, raw images were preprocessed using 14 filters to obtain corresponding derived images to reduce the impact of different ultrasound devices on features. Pyradiomics 2.1.2, an open-source software program, was utilized to take information out of all raw and derived images and convert it into quantitative features. The feature extraction taken was presented in Supplementary material 3.

### 2.6 Feature selection

After extracting all features, missing values for each feature were filled with means. Features in higher dimensions may have problems with low computational efficiency and overfitting (16, 25). Z-score normalization was used to eliminate dimensional differences in the data before feature selection. Features with ICC ≥ 0.8 were first selected, indicating that the feature was reproducible. Features with zero variance (i.e., features without any contribution to classification) were removed using variance filtering. Linear and nonlinear correlations between features and tags were captured using mutual information method, excluding features with maximal information coefficient (MIC) zero. Ultimately, the most valuable ultrasomics features were selected in combination with XGBoost embedding method.

### 2.7 Modeling and performance evaluation

5 machine learning algorithms, RF, XGBoost, SVM, DT, and LR, were used to construct clinical models, ultrasomics models, and clinical-ultrasomics combined models, respectively, for a total of 15.

Firstly, univariate analysis was performed for characteristics between EGFR^high^ and EGFR^low^ groups, including clinical data [Age, gender, maximum tumor diameter, tumor number, Child-Pugh(A/B/C), HbsAg/HbcAb (positive/negative), cirrhosis (yes/no), portal hypertension (yes/no), Edmondson-steiner grade, ALT, AST, TBIL, GGT, NLR, AFP] and qualitative imaging characteristics [lesion margin (clear/unclear), lesion echo (Hypo/Iso/hyper-echoic)]. Independent predictors were analyzed by including variables with *p* < 0.05 in univariate analysis into univariate and multivariate logistic regression. The above independent predictors were used to construct the clinical model by five machine learning algorithms.

The most valuable ultrasomics features extracted were used to construct the ultrasomics model through five machine learning algorithms. Finally, ultrasomics features were fused with clinical baseline features to build five combined models to investigate whether the accuracy of the model in predicting EGFR expression status could be improved.

**Figure 3:**
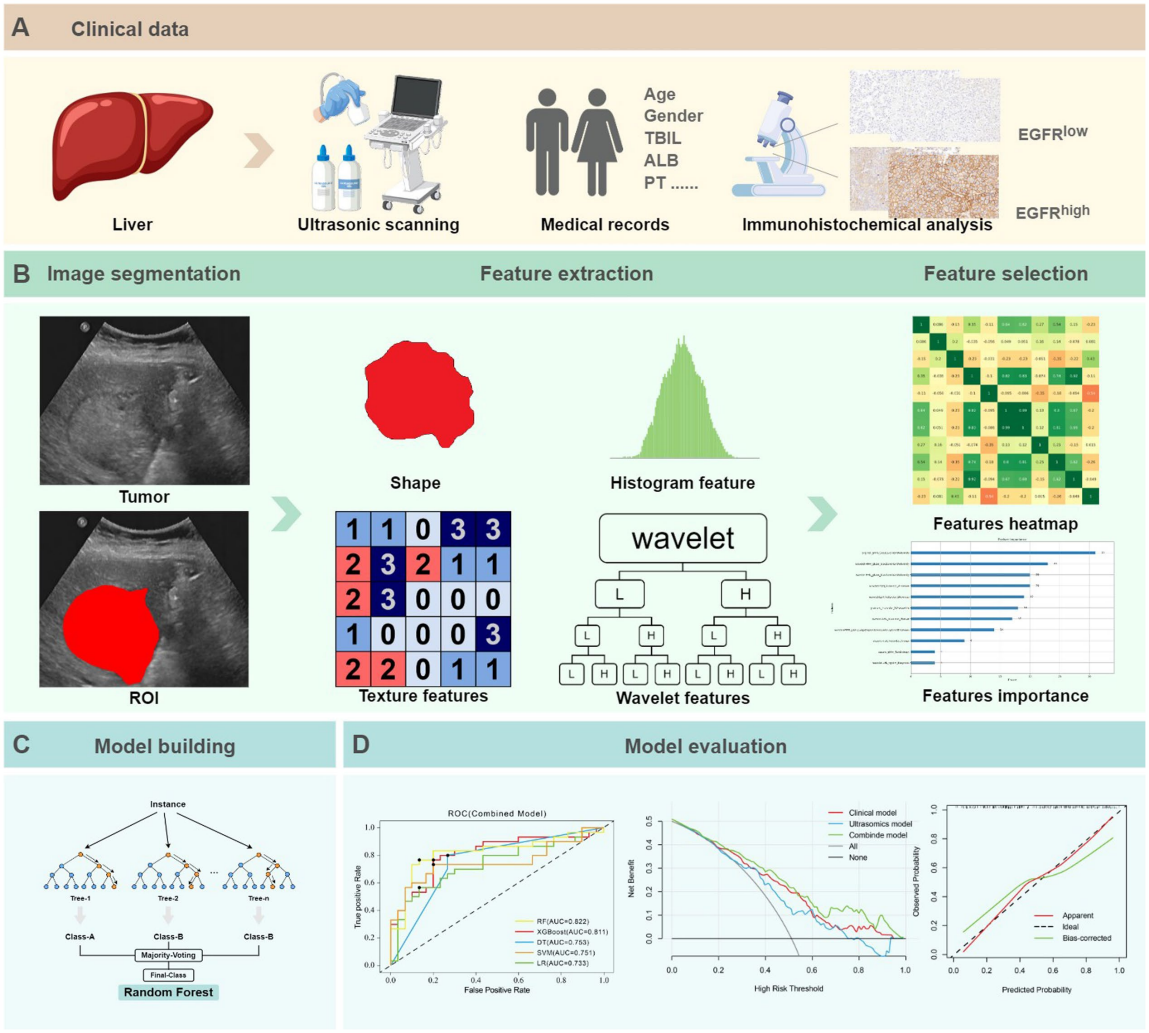
The ultrasomics workflow and study flowchart. **(A)** Clinical data. **(B)** Image segmentation, feature extraction and selection. **(C)** Model building. **(D)** Model evaluation.

The predictive ability of the model was evaluated through the area under the curve (AUC) value as well as its sensitivity, specificity, and accuracy. To evaluate the clinical practicability and efficiency of models, decision curve analysis (DCA) and calibration curve analyses were employed. Within the Python environment, the scikit-learn 0.23.2 package was used for both the model construction and evaluation. The workflow was illustrated as shown in Figure 3.

### 2.8 Statistical analysis

Statistical analysis was conducted using SPSS 26.0 and R 4.4.1. Continuous variables that were normally distributed were assessed using the independent sample *t*-test, while non-normal distributions were evaluated using a Mann–Whitney U test. Categorical variables were assessed using the chi-square test or Fisher’s exact test. A *p*-value of less than 0.05 was considered to indicate statistical significance.

## 3 Results

### 3.1 Clinical features

In the present study, 198 patients were included, with an average age of 57.07 ± 9.02 years, of whom 77.8% (*n* = 154) were male. There was no statistically significant difference in EGFR expression status and clinical baseline characteristics between the training and test dataset (*p* > 0.05). Table 1 summarized the clinical baseline characteristics of all patients.

**Table 1:** Patient clinical baseline characteristics of the training and test datasets.

Variables	Training dataset (*n* = 138)		Test dataset (*n* = 60)		*P*	*P* *
	EGFR^high^ (*n* = 70)	EGFR^low^ (*n* = 68)	EGFR^high^ (*n* = 30)	EGFR^low^ (*n* = 30)	0.925	
Age (years)^a^	55.96 ± 9.30	59.72 ± 8.69	53.77 ± 10.02	56.93 ± 6.39	0.077	0.005
Gender^b^					0.173	0.102
Male	54 (77.1)	57 (83.8)	19 (63.3)	24 (80.0)		
Female	16 (22.9)	11 (16.2)	11 (36.7)	6 (20.0)		
Child-Pugh^b^					0.078	0.648
A	58 (82.9)	55 (80.9)	28 (93.3)	27 (90.0)		
B or C	12 (17.1)	13 (19.1)	2 (6.7)	3 (10.0)		
Edmondson-steiner grade^b^					0.074	0.751
I	3 (4.3)	1 (1.5)	1 (3.3)	2 (6.7)		
II	46 (65.7)	44 (64.7)	16 (53.3)	13 (43.3)		
III	21 (30.0)	23 (33.8)	13 (43.3)	15 (50.0)		
Etiology of liver disease^b^					0.927	0.734
HBV positive	60 (85.7)	62 (91.2)	28 (93.3)	27 (90.0)		
HCV positive	8 (11.4)	2 (2.9)	0 (0)	3 (10.0)		
None or other	2 (2.9)	4 (5.9)	2 (6.7)	0 (0)		
Tumor number^b^					0.586	0.962
1	62 (88.6)	61 (89.7)	28 (93.3)	27 (90.0)		
≥2	8 (11.4)	7 (10.3)	2 (6.7)	3 (10.0)		
Margins^b^					0.811	0.013
Obscure	32 (45.7)	23 (33.8)	17 (56.7)	8 (26.7)		
Clear	38 (54.3)	45 (66.2)	13 (43.3)	22 (73.3)		
Echogenicity^b^					0.837	0.693
Hypo-echoic	42 (60.0)	33 (48.5)	14 (46.7)	16 (53.3)		
Iso-echoic	9 (12.9)	9 (13.2)	3 (10.0)	5 (16.7)		
Hyper-echoic	19 (27.1)	26 (38.2)	13 (43.3)	9 (30.0)		
Portal hypertension^b^					0.719	0.131
Positive	41 (58.6)	45 (66.2)	17 (56.7)	22 (73.3)		
Negative	29 (41.4)	23 (33.8)	13 (43.3)	8 (26.7)		
Cirrhosis^b^					0.187	0.653
Yes	61 (87.1)	62 (91.2)	29 (96.7)	28 (93.3)		
No	9 (12.9)	6 (8.8)	1 (3.3)	2 (6.7)		
Maximum diameter (mm)^c^	37.00 (26.75,54.50)	39.00 (24.25,57.50)	31.50 (19.00,47.50)	47.50 (32.00,78.00)	0.548	0.082
ALT (U/L)^c^	31.30 (21.10,45.93)	29.80 (16.98,59.20)	24.15 (17.32,50.85)	26.80 (16.17,52.17)	0.380	0.838
AST (U/L)^c^	30.10 (22.65,44.30)	35.50 (23.47,47.77)	33.50 (22.05,56.80)	27.70 (21.02,54.30)	0.712	0.714
GGT (U/L)^c^	48.20 (28.75,116.47)	47.25 (30.40,95.10)	46.55 (28.50,73.80)	57.20 (30.15,114.80)	0.879	0.442
AFP (ng/ml)^c^	91.82 (7.42,1467.75)	29.97 (5.06,408.30)	69.09 (15.14,1063.50)	34.41 (6.27,285.55)	0.688	0.041
NLR^c^	2.38 (1.50,2.94)	2.12 (1.47,2.83)	2.62 (1.81,3.28)	1.82 (1.56,2.13)	0.896	0.031

EGFR^high^, EGFR high expression; EGFR^low^, EGFR low expression; ALT, alanine aminotransferase; AST, aspartate aminotransferase; GGT, glutamyl transpeptidase; AFP, serum alpha-fetoprotein; NLR, neutrophil-to-lymphocyte ratio; *P* represents the comparison of features between training and test datasets; *P* * represents the comparison of features between dataset with high and low EGFR expression.

a Data are $\bar{x\;}$ ±SD.

b Data are *n* (%), *N* indicates the number of participants for which data is available.

c Data are median (25th–75th percentile).

### 3.2 Feature extraction and selection

A total of 1,409 features were taken out of the original and derived images. 285 features with ICC less than 0.8 were excluded. Using variance filtering and mutual information techniques, the remaining 1,124 features, 16 features with 0 in order variance, and 495 features with nil mutual information characteristics were eliminated. 602 features were further excluded using the embedded method of XGBoost, ultimately identifying 11 of the most valuable ultrasomics features, including original, shape, first-order, second-order texture, square, exponential, gradient, and higher-order (wavelet features, etc.). The feature extraction and selection process were detailed in Supplementary materials 3, 4.

### 3.3 Predictive performance of clinical models

**Figure 4:**
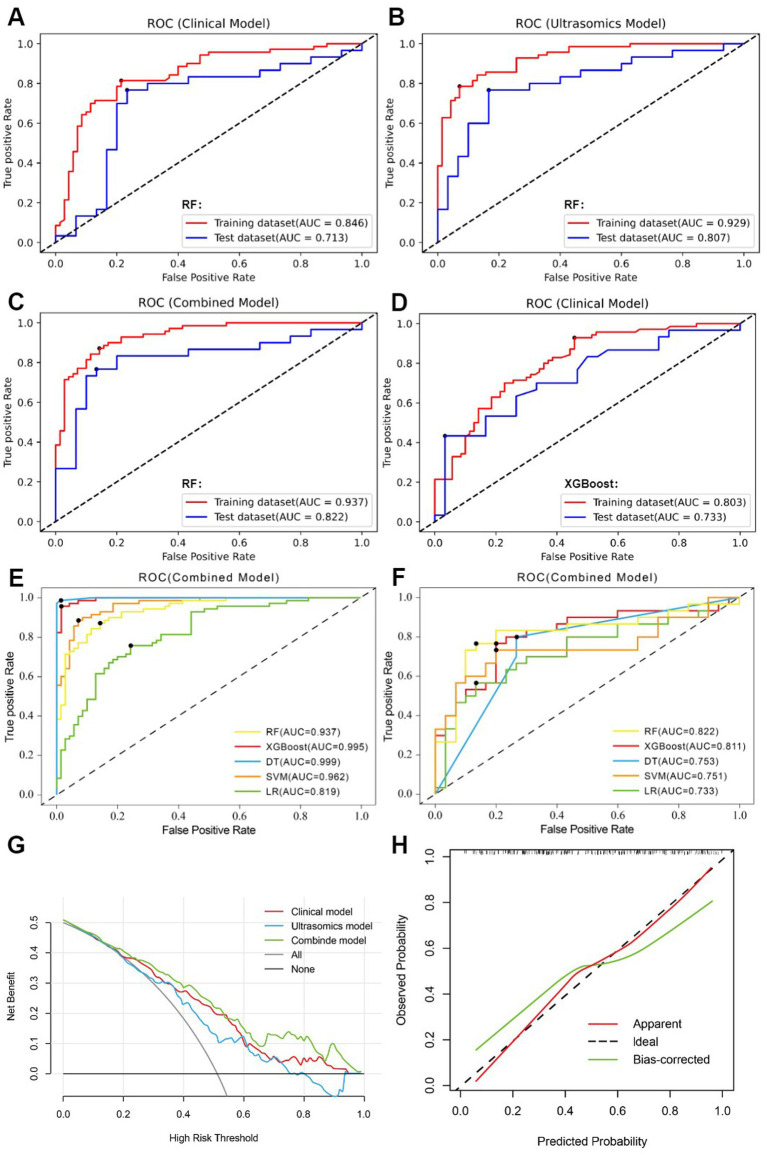
**(A)** clinical model of the RF algorithm. **(B)** ultrasomics model of the RF algorithm. **(C)** combined model of the RF algorithm. **(D)** clinical model of the XGBoost algorithm. **(E)** AUC comparison of five machine learning algorithms constructed as combined models in the training dataset. **(F)** AUC comparison of five machine learning algorithms constructed as combined models in the test dataset. **(G)** DCA of three RF algorithm models. **(H)** The calibration curve for the RF algorithm’s combined model.

**Figure 5:**
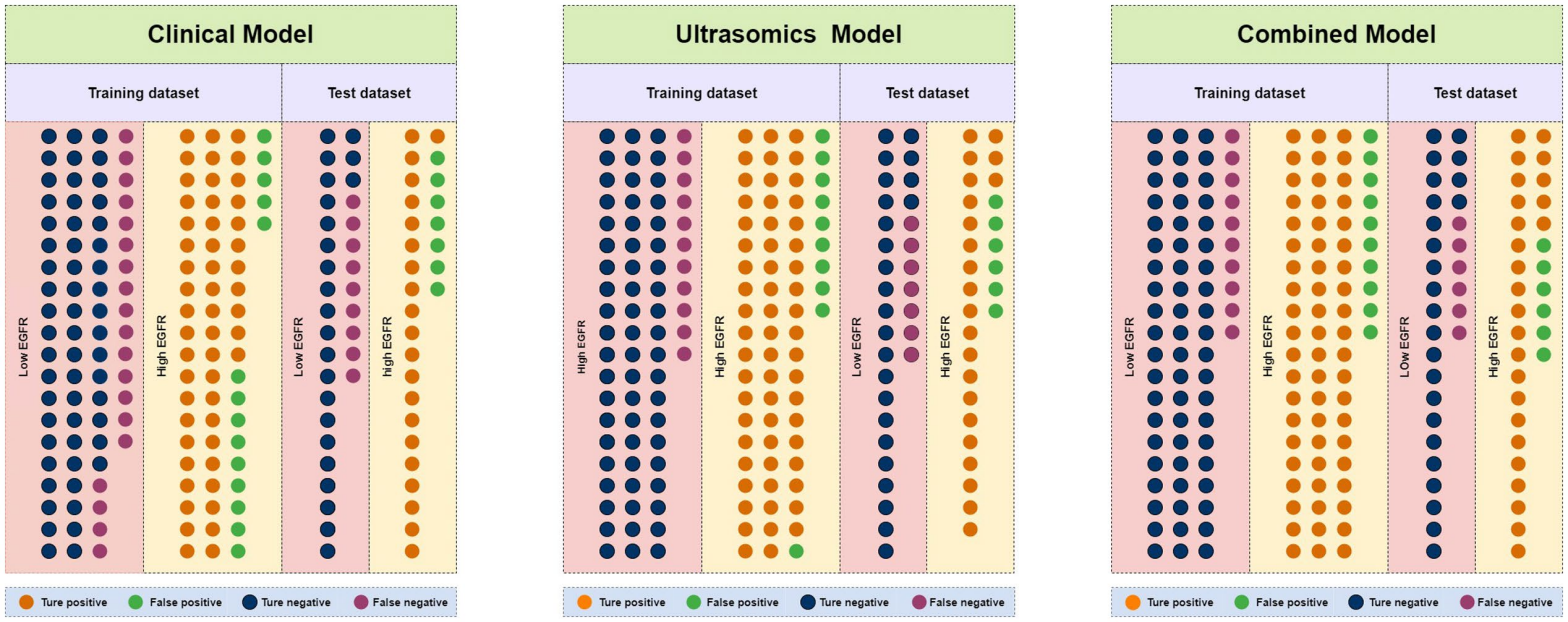
Number of true positive, false positive, true negative, and false negative events in the training and test dataset for clinical models, ultrasomics models, and combined models of RF algorithm.

Through univariate analysis of characteristics between EGFR^high^ and EGFR^low^ groups, there were significant differences in three clinical data (age, AFP, NLR) and one qualitative imaging feature (lesion margin) (*p* < 0.05). Univariate and multiple logistic regression analyses of these variables showed age (*OR* = 0.958, 95% CI 0.926–0.991, *p* = 0.013) and focus margin characteristics (*OR* = 2.114, 95% CI 1.164–3.839, *p* = 0.014) as independent predictors (Table 2). The above variables were used to construct the clinical model using five machine learning algorithms, and the clinical model with good predictive performance was RF and XGBoost classifier. AUC in the test dataset was 0.713 (95%CI, 0.582–0.823) and 0.733 (95%CI, 0.603–0.839), respectively (Figures 4A,D), sensitivity was 0.700 and 0.700, specificity was 0.767 and 0.633, and accuracy was 0.733 and 0.667, respectively. The clinical model of RF classifier was higher than the clinical model of XGBoost classifier in specificity and accuracy, showed that the RF classifier established a clinical model with good predictive performance. In the test dataset, 21 of 30 EGFR^high^ patients and 23 of 30 EGFR^low^ patients were identified by the clinical model of RF classifier (Figure 5).

**Table 2:** Univariate and multivariate assessments of variables related to EGFR expression status.

	Univariate		Multivariate	
	OR (95%CI)	*P*	OR (95%CI)	*P*
Age	0.955 (0.923–0.987)	0.006	0.958 (0.926–0.991)	0.013
Margins	2.077 (1.164–3.704)	0.013	2.114 (1.164–3.839)	0.014
AFP	1.000 (1.000–1.000)	0.085		
NLR	0.992 (0.938–1.048)	0.771		

AFP, serum alpha-fetoprotein; NLR, neutrophil-to-lymphocyte ratio neutrophil-to-lymphocyte ratio; CI, confidence interval; OR, odds ratio.

### 3.4 Predictive performance of ultrasomics and combined models

11 most valuable ultrasomics features were analyzed and ultrasomics models were built by five machine learning algorithms. The results showed that the Ultrasomics model of RF classifier performed well in predicting EGFR expression in HCC patients, with AUC of 0.929 (95%CI, 0.874–0.966) and 0.807 (95%CI, 0.684–0.897) (Figure 4B), sensitivity of 0.843 and 0.767, specificity of 0.857 and 0.800, and accuracy of 0.850 and 0.783 in the training and test dataset, respectively. In the test dataset, 23 of 30 EGFR^high^ patients and 24 of 30 EGFR^low^ patients were identified by the ultrasomics model of RF classifier (Figure 5).

Finally, the predictive performance of the model was further optimized by fusing ultrasomics features with clinical baseline features to build a combined model, and in the test dataset, the AUC of the combined model established by the five machine learning algorithms was RF (0.822), XGboost (0.811), DT (0.753), SVM (0.751), and LR (0.733), and the combined model predictive performance of RF classifier was better than that of the other models (Figures 4C,F). In the test dataset, 25 of 30 EGFR^high^ patients and 24 of 30 EGFR^low^ patients were identified by the combined model of RF classifier (Figure 5). Performance evaluation measures for the model were shown in Table 3.

**Table 3:** Predicted results of clinical models, ultrasomics models, and combined models.

Model	Training dataset (*n* = 138)					Test dataset (*n* = 60)				
	SEN	SPE	ACC	AUC (95%CI)	*P*	SEN	SPE	ACC	AUC (95%CI)	*P*
Clinical										
RF	0.729	0.800	0.764	0.846 (0.776–0.902)	<0.0001	0.700	0.767	0.733	0.713 (0.582–0.823)	0.0032
XGBoost	0.714	0.729	0.721	0.803 (0.727–0.865)	<0.0001	0.700	0.633	0.667	0.733 (0.603–0.839)	0.0004
SVM	0.843	0.886	0.864	0.945 (0.894–0.977)	<0.0001	0.667	0.733	0.700	0.707 (0.575–0.817)	0.0028
DT	0.886	0.757	0.821	0.928 (0.872–0.965)	<0.0001	0.700	0.667	0.683	0.713 (0.582–0.823)	0.0012
LR	0.714	0.729	0.721	0.790 (0.713–0.854)	<0.0001	0.667	0.700	0.683	0.694 (0.562–0.807)	0.0061
Ultrasomics										
RF	0.843	0.857	0.850	0.929 (0.874–0.966)	<0.0001	0.767	0.800	0.783	0.807 (0.684–0.897)	<0.0001
XGBoost	0.729	0.757	0.743	0.818 (0.744–0.878)	<0.0001	0.733	0.700	0.717	0.740 (0.611–0.845)	0.0003
SVM	0.971	0.971	0.971	0.995 (0.965–1.000)	<0.0001	0.733	0.700	0.717	0.747 (0.618–0.850)	0.0001
DT	0.971	0.843	0.907	0.979 (0.939–0.996)	<0.0001	0.733	0.667	0.700	0.690 (0.557–0.803)	0.0045
LR	0.614	0.629	0.621	0.655 (0.570–0.733)	0.0008	0.667	0.700	0.683	0.690 (0.557–0.803)	0.0074
Combined										
RF	0.857	0.857	0.857	0.937 (0.884–0.971)	<0.0001	0.833	0.800	0.817	0.822 (0.702–0.909)	<0.0001
XGBoost	0.957	0.971	0.964	0.995 (0.964–1.000)	<0.0001	0.800	0.733	0.767	0.811 (0.689–0.901)	<0.0001
SVM	0.857	0.929	0.893	0.962 (0.916–0.987)	<0.0001	0.733	0.800	0.767	0.751 (0.623–0.854)	0.0002
DT	0.986	0.986	0.986	0.999 (0.972–1.000)	<0.0001	0.733	0.733	0.733	0.753 (0.625–0.856)	<0.0001
LR	0.714	0.757	0.736	0.819 (0.746–0.879)	<0.0001	0.700	0.700	0.700	0.733 (0.603–0.839)	0.0005

AUC, Area under the receiver operating characteristic curve; SEN, sensitivity; SPE, specificity; ACC, accuracy; *P* < 0.05 indicates a significant difference in the identification of EGFR expression status.

Among the five machine learning algorithms, the three models of RF classifier demonstrated the best prediction performance. The combined model, however, showed superior clinical net benefit, indicating greater applicability in clinical practice (Figure 4G). Additionally, its calibration curve demonstrated a sufficient degree of agreement between the predicted EGFR expression status and actual results (Figure 4H), showing more stable prediction performance.

## 4 Discussion

HCC is the most common type of liver cancer, with poor prognosis and 5-year survival rate of 18%, and is one of several malignancies with high fatality rate worldwide (3). Multiple molecular biomarkers such as EGFR, ki-67, VEGF, P53 have been identified as the main factors involved in HCC progression and affecting prognosis (24, 26–28), of which EGFR is often highly expressed in HCC and is involved in proliferation, invasion and metastasis of tumor cells, resulting in poor prognosis of HCC (29–31). EGFR was found highly expressed in 50.5% of HCC patients in the present study, which is close to previous findings (8, 10).

Recently, some studies have found that EGFR^high^ in liver cancer cells is associated with resistance to targeted agents such as Lenvatinib (32, 33). Lenvatinib has been approved by Food and Drug Administration (FDA) as first-line treatment for aHCC. However, the objective response rate was only 24.1%, indicating lenvatinib needs to be combined with other drugs to improve its clinical benefit (34, 35). A study conducted by Jin et al. (12) found that lenvatinib, in HCC patients with high EGFR expression, induced feedback activation of EGFR and its downstream signaling pathways by inhibiting FGFR, leading to HCC cells still having strong proliferative capabilities. EGFR inhibitors could block the feedback-activated signaling pathways, enhancing the antitumor effect. A clinical trial initiated by Renji Hospital (NCT04642547) recruited aHCC patients with high EGFR expression, using a combination treatment of lenvatinib and EGFR inhibitors, and the clinical response rate reached 50% (12, 15, 36). Therefore, HCC patients with noninvasive identification EGFR^high^ are important conditions for therapeutic management.

In the present study, we compared the predictive performance of five machine learning algorithms constructed clinical models, ultrasomics models, and combined models for noninvasive prediction of EGFR expression in HCC patients. The findings demonstrated that the five machine learning algorithms’ ultrasomics features could successfully differentiate between the EGFR expression status of HCC patients in the training and test datasets (*p* < 0.05), and that the ultrasomics model and the combined model constructed with an RF classifier outperformed the others in terms of predictive performance (Figures 4E,F; Table 3).

**Figure 6:**
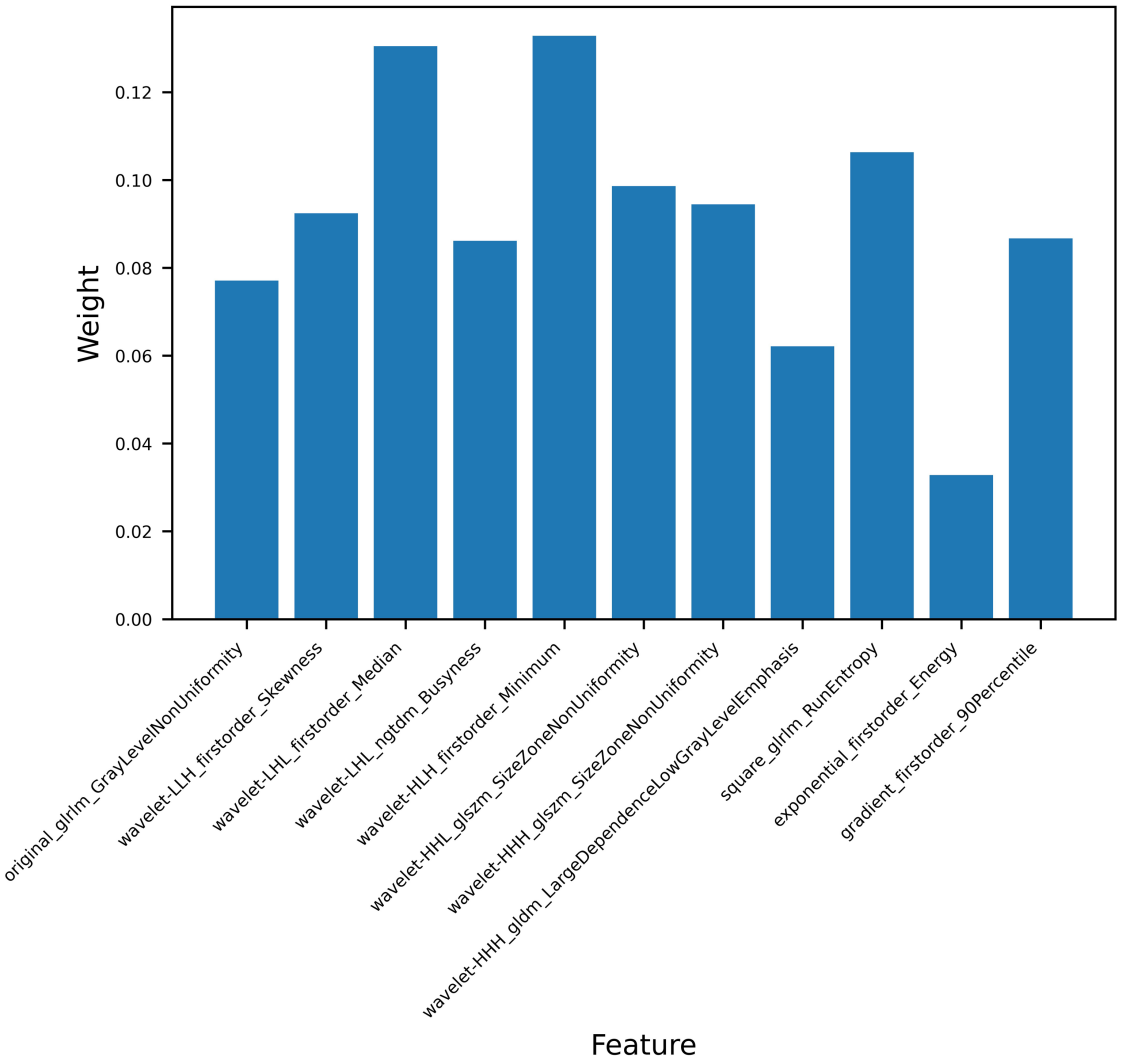
Weighting coefficients of 11 of the ultrasomics features.

The ultrasomics models developed according to five machine learning algorithms showed good predictive ability, with the RF classifier having the best predictive performance, and the AUC of the training and test datasets increased from 0.846 (95%CI, 0.776–0.902) and 0.713 (95%CI, 0.582–0.823) to 0.929 (95%CI, 0.874–0.966) and 0.807 (95%CI, 0.684–0.897), respectively, for the clinical model. The improvement in predictive performance is because ultrasomics can extract more features from images that are associated with tumor heterogeneity and assess them quantitatively (17, 37, 38). Wu et al. developed an radiomics model based on energy-enhanced CT to predict EGFR expression status in peripheral lung cancer (39). Features such as the arterial phase Laplace of Gaussian Filter Glszm Small Area Low Gray Level emphasis and wavelet HHL gray level co-occurrence matrix (GLCM) MCC, and the venous phase wavelet LHL first-order root mean square were extracted. A multiphasic model established based on the features from both phases was found to have good predictive performance (AUC 0.950). The results show that imaging features, especially higher-order features, can better predict the expression of EGFR. In this study, 7 of the 11 best features are high-order features obtained by wavelet filtering, which indicates that higher-order features can obtain more EGFR-related features, which is the same as previous research results. The seven wavelet transform features were primarily derived from gray-level size-zone matrix (GLSZM) features and first-order features. GLSZM was used to describe the spatial distribution of gray level values and the information about the size of regions in the image, while First-order features mainly described the symmetry, uniformity, and distribution changes of image voxels’ intensity (38, 40). Among the 11 features, the wavelet HLH first-order Minimum, wavelet LHL first-order Median, square gray-level run length matrix (GLRLM) RunEntropy, and wavelet HHL GLSZM Size Zone NonUniformity features were found to have the highest coefficients (Figure 6). The square feature involves squaring each pixel value in the image to enhance the contrast of gray level values (41). These results indicate that wavelet transform features could increase the predictive value of radiomic features, being more sensitive to the identification of tumor heterogeneity and can be used to predict the EGFR expression status (42). When clinical baseline characteristics were incorporated into the ultrasomics model, the RF machine learning combined model demonstrated better predictive performance, with AUCs of 0.937 (95%CI, 0.884–0.971) for the training dataset and 0.822 (95%CI, 0.702–0.909) for the test dataset, showing a slight improvement over the ultrasomics model alone. Therefore, the ultrasomics model and combined model established by the RF classifier can better predict the EGFR expression status in HCC patients.

Qualitative characteristics of lesion margin on ultrasound images analyzed by multivariate logistic regression may serve as independent predictors of EGFR expression status. Unclear lesion boundary is a risk factor, which may be related to EGFR^high^ more aggressive and proliferative ability leading to changes in lesion morphology (40, 43). In univariate analysis, there was a significant difference between groups in serum AFP and NLR (*p* < 0.05), and correlation analysis showed that the correlation coefficients (*r*) between AFP and NLR and EGFR expression status were 0.146 and 0.154, respectively (*p* < 0.05) (Supplementary Figure S3), indicating that serum AFP and NLR had a low correlation with EGFR expression status. Fan et al. predicted VEGF expression in HCC patients based on MRI imageomics profiles, and multivariate logistic regression analysis showed that AFP, NLR, and irregular lesion boundaries were independent predictors, and AUC of the clinical model and imageomics model in the training and test dataset were 0.709, 0.725; 0.892, 0.800, respectively (44). The AUC of the RF machine learning clinical model in this study was 0.846 (95%CI, 0.776–0.902) and 0.713 (95%CI, 0.582–0.823) in the training and test dataset, respectively, and compared to the ultrasomics model, the prediction accuracy was lower (AUC 0.929, 0.807). These data suggested that clinical baseline characteristics have limited predictive power for molecular tumor phenotypes.

Due to the limitations of EGFR immunohistochemical testing in the patients, in this present study, there was just a single center involved and lacked external validation to enhance the generalizability of the predictive model. Another limitation of the study was that manual segmentation was time-consuming and inefficient. Thus, in the future, there is a need for convenient, efficient, and repeatable automatic segmentation software that must be clinically validated. Third, the baseline images in this experiment were taken from different ultrasound instruments. Although the image is preprocessed before feature extraction, there may be confounding factors that affect the results. Finally, the present study only analyzed gray-scale ultrasound and did not assess in conjunction with contrast-enhanced ultrasound, elastography, and other imaging modalities. In our future work, multimodal imaging radiomics will be explored for the EGFR expression levels in HCC patients.

## 5 Conclusion

In conclusion, the construction of ultrasomics based on gray-scale ultrasound images by five machine learning algorithms can be used as noninvasive and effective diagnostic tools to predict EGFR expression status in HCC patients. Furthermore, the ultrasomics model and combined model established by RF classifier have the best predictive performance. The present study will provide a new noninvasive method for noninvasive prediction and precise treatment of EGFR expression status in patients with aHCC.

## Data Availability

The raw data supporting the conclusions of this article will be made available by the authors, further inquiries can be directed to the corresponding author.
