# Supplementary material for: Machine learning based ultrasomics noninvasive predicting EGFR expression status in hepatocellular carcinoma patients
Source: Front Med (Lausanne). 2024 Nov 19;11:1483291. doi: 10.3389/fmed.2024.1483291 (PMC11609927; doi:10.3389/fmed.2024.1483291)
Supplement: Supplementary file 1 [file Data_Sheet_1.docx]

Supplementary Material

**Supplementary material 1**. Preparation of EGFR immunohistochemical sections

The resected specimen tissues were fixed in 10% neutral formalin, processed, embedded in paraffin, cut into 4-μm-thick sections, deparaffinized, and hydrated. Then, immunohistochemical staining was performed with a rabbit monoclonal antibody EGFR antibody reagent (clone SP111). The secondary antibodies were added and incubated at 4°C overnight. Finally, they were stained with 3,3′-diaminobenzidine (DAB) and counterstained with hematoxylin.

**Supplementary material 2**. Ultrasonic equipment and inspection requirements

Ultrasound examination equipment included GE Logiq E20, GE Vivid E9, HIVISION Ascendus, HIALOK ProSound A5, Philips EPIQ 7, or Philips EPIQ 5. The ultrasound probes were all C75, with frequencies ranging from 1 to 5 MHz. The image settings for each examination, such as time gain compensation, focal point position, dynamic range, etc., were optimized according to the manufacturer's recommendations. All patients were fasted for more than 8 hours before the examination and were placed in a supine position for the acquisition of liver lesion images.

**Supplementary material 3**. Feature extraction

In the present study, Pyrodiomics v.2.1.2 software package was used to extract all image-omics features from one original image and 14 derived images, such as Wavelet (wavelet-LLH, wavelet-LHL, wavelet-LHH, wavelet-HLH, wavelet-HLL, wavelet-LLL, wavelet-HHL, wavelet-HHH), Square, SquareRoot, Logarithm, Exponential, Gradient, LocalBinaryPattern 2D. In addition to shape features, features for all categories were taken directly from the original and derived images.

**Supplementary material 4**. Feature selection

The intraclass correlation coefficient (ICC) screening method, variance threshold method, mutual information method, and embedding method combined with the limit gradient lifting method (XGBoost) were used for feature screening. Finally, 11 ultrasomics tags were selected for model building, as shown in the figure below:

| No. | Feature Name | Coefficient |
| --- | --- | --- |
| 1 | original_glrlm_GrayLevelNonUniformity | 0.0771189 |
| 2 | wavelet-LLH_firstorder_Skewness | 0.09241955 |
| 3 | wavelet-LHL_firstorder_Median | 0.13047993 |
| 4 | wavelet-LHL_ngtdm_Busyness | 0.08614925 |
| 5 | wavelet-HLH_firstorder_Minimum | 0.13281915 |
| 6 | wavelet-HHL_glszm_SizeZoneNonUniformity | 0.0986009 |
| 7 | wavelet-HHH_glszm_SizeZoneNonUniformity | 0.0944775 |
| 8 | wavelet-HHH_gldm_LargeDependenceLowGrayLevelEmphasis | 0.06212676 |
| 9 | square_glrlm_RunEntropy | 0.10632417 |
| 10 | exponential_firstorder_Energy | 0.03280341 |
| 11 | gradient_firstorder_90Percentile | 0.08668038 |


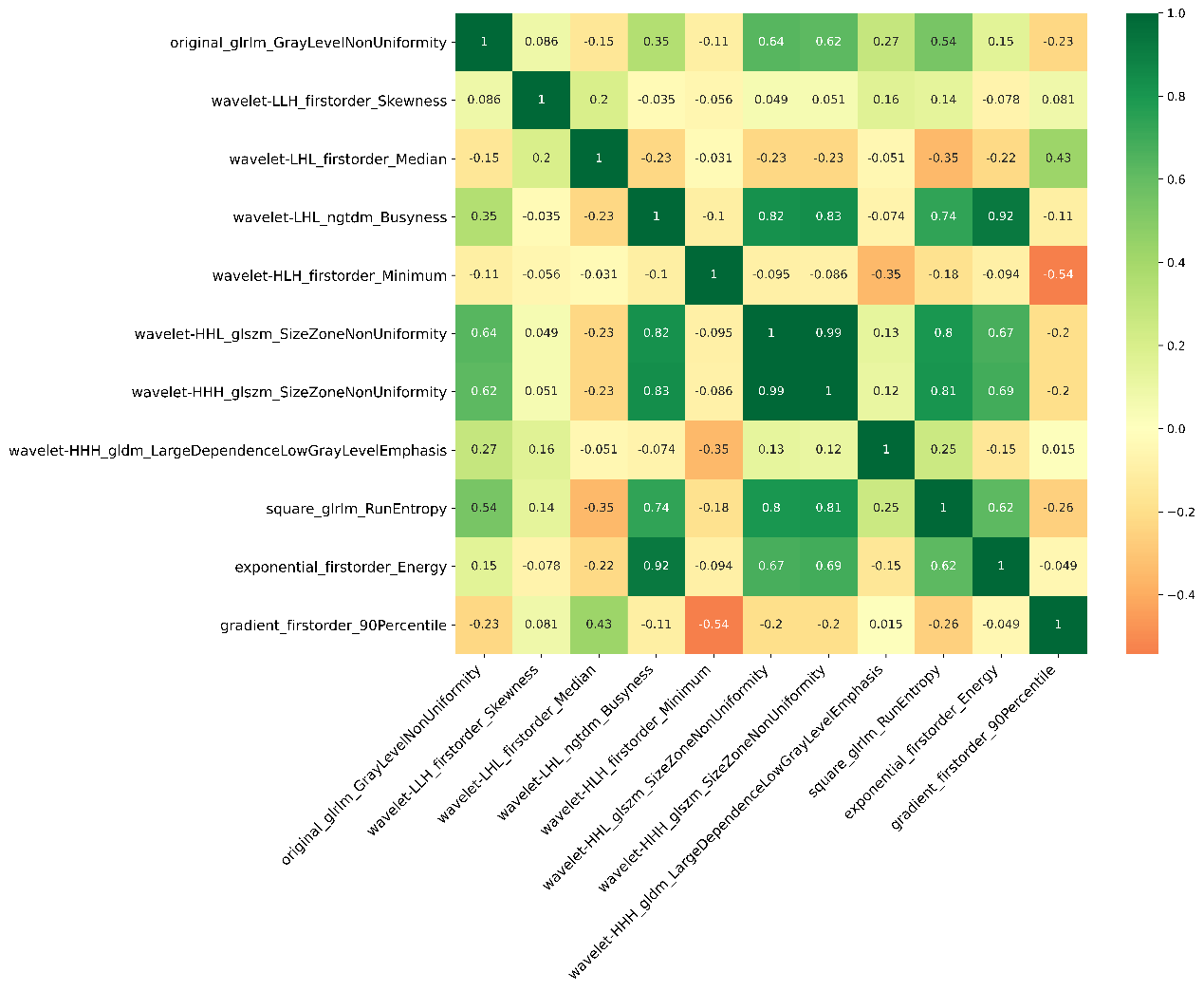


**Supplementary Figure 1.** The heatmap of features shows the correlation between 11 significant image-omics labels extracted from the training dataset by color. The redder the color, the higher the correlation. Conversely, the greener the color, the lower the correlation.


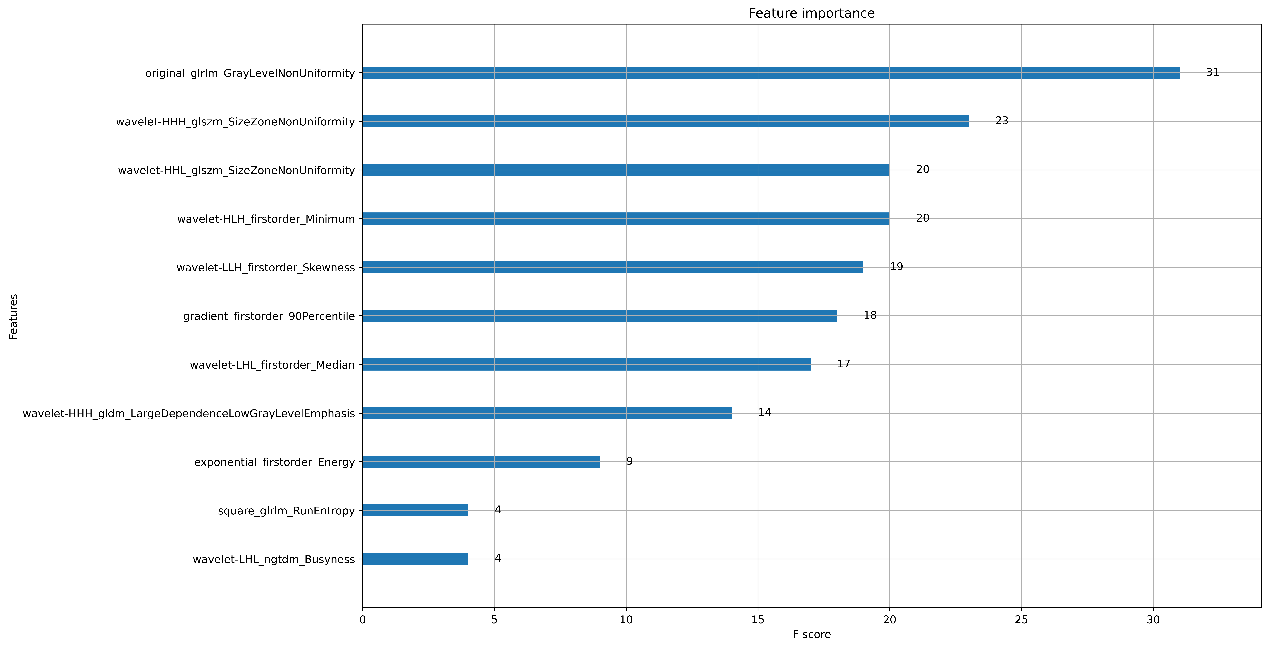
 **Supplementary Figure 2.** The feature importance plot displayed the 11 significant radiomic labels and their corresponding feature coefficients.

|  |  |  | label | AFP |
| --- | --- | --- | --- | --- |
| Spearman | label | *r* | 1.000 | 0.146 |
|  |  | sig.(2-tailed) |  | 0.041 |
|  |  | N | 198 | 198 |
|  |  |  | label | NLR |
|  | label | *r* | 1.000 | 0.154 |
|  |  | sig.(2-tailed) |  | 0.031 |
|  |  | N | 198 | 198 |

**Supplementary Figure 3.** Statistical analysis of the correlation between AFP, NLR, and EGFR expression status.
